# A data-driven approach for mitigation of fecal pathogen infections from unsafe WASH practices

**DOI:** 10.1016/j.onehlt.2026.101317

**Published:** 2026-01-03

**Authors:** Achara Taweesan, Thammarat Koottatep, Thongchai Kanabkaew, Rathanit Sukthanapirat, Chongrak Polprasert

**Affiliations:** a Department of Environmental Science, Faculty of Science, Ramkhamhaeng University, Bangkok, Thailand; b School of Environment, Resources and Development, Asian Institute of Technology, Pathumthani, Thailand; c Occupational and Environmental Health Program, Faculty of Public Health, Thammasat University, Pathumthani, Thailand; d Department of Civil and Environmental Engineering, Faculty of Science and Engineering, Kasetsart University Chalermphrakiat Sakon Nakhon Province Campus, Sakon Nakhon, Thailand; e Department of Civil Engineering, Faculty of Engineering, Thammasat University, Pathumthani, Thailand

**Keywords:** Fecal pathogen infections, Fecal sludge management, Public health interventions, Response surface methodology (RSM), Sustainable development goal 6 (SDG 6), Water, sanitation, and hygiene (WASH)

## Abstract

Fecal pathogen infections remain a major public health challenge in low- and middle-income countries, where unsafe water, inadequate sanitation, and poor hygiene persist. Northeastern Thailand continues to experience a high burden of helminth infections linked to deficient water, sanitation, and hygiene (WASH) conditions. Evidence-based identification of combined WASH thresholds is needed to support effective interventions and progress toward Sustainable Development Goal 6.

A cross-sectional study was conducted among 520 households across 18 communities in Tongkhop city, Sakon Nakhon Province, Thailand. Primary data from household surveys, key-informant interviews, and field observations were integrated with disease-surveillance records. A multilevel generalized linear model was applied to assess associations between WASH indicators and infection prevalence. Model-predicted outcomes were further analyzed using response surface methodology to quantify nonlinear interactions and identify optimal WASH thresholds.

Infection prevalence exceeded 40 cases per 1000 population in communities relying on untreated water, infrequent fecal-sludge emptying, and low hygiene compliance. Substantial risk reductions were observed when household water treatment exceeded 40 %, fecal-sludge emptying frequency reached 35–40 %, and handwashing compliance before meals surpassed 80 %, while raw-fish consumption remained below 50 %. The response-surface analysis revealed clear nonlinear synergies among WASH components, indicating that coordinated improvements were more effective than isolated interventions.

This study demonstrates that integrating multilevel modeling with response surface analysis enables quantitative identification of critical WASH thresholds for reducing fecal-pathogen infections. The findings highlight the importance of coordinated improvements in water safety, sanitation management, and hygiene behaviors to mitigate environmental transmission pathways. These results provide actionable, data-driven guidance for public health planning and support the environmental dimension of the One Health framework in advancing SDG 6.

## 1 Introduction

Fecal pathogen infections represent a persistent global public health challenge, particularly in low- and middle-income countries where inadequate water, sanitation, and hygiene (WASH) practices remain widespread [18,27,41]. Unsafe drinking water, poor sanitation, and insufficient hygiene contribute to diseases such as diarrhea and helminth infections, disproportionately affecting vulnerable populations [10,31]. According to the World Health Organization (WHO), over 1.5 billion people, approximately 24 % of the global population, are infected with soil-transmitted helminths, with the highest prevalence in tropical and subtropical regions, including sub-Saharan Africa and Southeast Asia [28].

In Thailand, helminth infections remain a pressing public health concern, particularly in rural and semi-urban areas of the northeastern region, where infection rates are highest. A national survey reported a helminthiasis prevalence of 20 % among 16,000 individuals, with provinces such as Khon Kaen, Sakon Nakhon, Surin, and Ubon Ratchathani experiencing the highest burden [13,43]. The survey identified over 14 species of helminths, with hookworms being the most prevalent (10 %), followed by *Opisthorchis viverrini* (5 %), a parasitic liver fluke associated with cholangiocarcinoma, a deadly bile duct cancer. Other common infections included *Ascaris lumbricoides* (roundworm) and *Trichuris trichiura* (whipworm).

Inadequate hygiene further exacerbates the disease burden, contributing to approximately 25 % of preventable infections globally [40]. Evidence suggests that simple interventions, such as handwashing with soap, can reduce fecal pathogen infections by up to 30 %, highlighting their cost-effectiveness and scalability [44]. Nevertheless, northeastern Thailand faces compounded WASH challenges due to limited access to safe water and inefficient sanitation systems, particularly in provinces such as Sakon Nakhon, Yasothon, and Amnat Charoen. Poor environmental sanitation results in contamination of shared water resources and food, increasing exposure to pathogens from agricultural runoff and domestic wastewater discharge [9,13].

These interrelated challenges underscore the importance of the One Health framework, particularly its environmental dimension, which highlights the linkage between human health and environmental quality. In northeastern Thailand, ineffective fecal sludge management, agricultural runoff, and wastewater discharge perpetuate fecal–oral transmission cycles that threaten community health. Although the present study does not include direct animal health parameters, it integrates the environmental dimension of One Health by analyzing how sanitation and ecological factors jointly influence infection risks. Evidence from recent One Health sanitation research shows that improved fecal-waste containment can reduce the environmental pathways that support zoonotic transmission. Expanded sanitary toilet coverage, for example, significantly lowered the incidence of *brucellosis*, *echinococcosis*, and *leptospirosis* by interrupting pathogen cycles shared between humans, animals, and contaminated environments [15,18,46]. This decreases environmental pathogen loads that otherwise allow zoonotic agents to circulate among animal hosts and spill over to humans, as demonstrated in the provincial-level reductions in zoonotic disease incidence observed in China [46]. Effective mitigation therefore requires coordinated efforts across public health, environmental, and local administrative sectors to strengthen water safety, sanitation infrastructure, and environmental protection. Integrating these elements enhances both community health and ecosystem resilience, aligning with Sustainable Development Goal 6 (Clean Water and Sanitation).

Previous research has often investigated individual WASH components, such as water access, sanitation, or hygiene, independently. However, recent studies increasingly employ integrated and systems-based approaches that examine their combined effects on sustainability and intervention efficacy [16,20]. For example, although water treatment interventions such as filtration and disinfection effectively reduce waterborne disease transmission [5], many sanitation studies still overlook the interdependencies between water quality, hygiene practices, and waste management [28]. Such fragmented analyses fail to capture the nonlinear, synergistic relationships that determine overall WASH effectiveness.

To address this gap, the present research applies a data-driven approach using Response Surface Methodology (RSM) to quantify how combinations of WASH conditions influence fecal pathogen infections in northeastern Thailand. RSM is a robust statistical technique designed to model complex, nonlinear interactions among multiple variables and identify optimal intervention thresholds [37,42]. By integrating RSM with multilevel modeling, this study identifies quantitative WASH thresholds that yield maximum reductions in infection prevalence. The findings aim to inform policy design, enhance intersectoral coordination, and support data-based prioritization of WASH interventions consistent with the One Health framework and SDG 6.

## 2 Materials and methods

### 2.1 Study design and approach

**Fig. 1:**
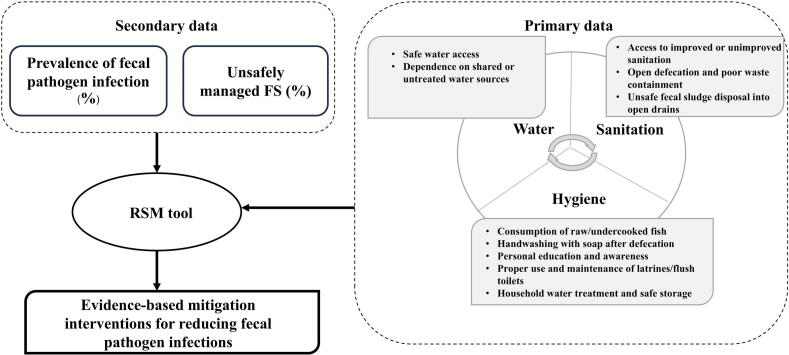
Methodology framework.

This study adopted an integrated multilevel methodology within the environmental dimension of the One Health framework. The research design combined primary and secondary datasets to assess how environmental sanitation, water management, and hygiene behaviors jointly influence infection risk. Collaboration with local public health and environmental agencies ensured the ecological relevance and policy applicability of the findings. A predictive response-surface analysis approach was further applied to develop quantitative models for mitigating fecal pathogen infections (Fig. 1).

The analysis examined the relationships between WASH practices and infection prevalence across 18 communities situated in high-risk areas of northeastern Thailand. Secondary data were analyzed to establish baseline statistics on fecal-pathogen infections and the proportion of unsafely managed fecal sludge, while primary data were collected from 520 households distributed across these communities. Approximately 75 % of the primary dataset was obtained through structured household questionnaires, and the remaining 25 % through key-informant interviews and structured household observations used to verify and supplement missing WASH information.

Key informants comprised village heads and assistant village heads (25; 19 %), public health officers (22; 17 %), village health volunteers (18; 14 %), local environmental-sanitation officers (24; 18 %), community water-supply operators or committee members (9; 7 %), and other relevant stakeholders (32; 25 %). These informants were purposively selected based on their direct roles in community-level WASH management and their familiarity with household sanitation, water-supply systems, and hygiene behaviors. This diversity of perspectives provided a comprehensive contextual understanding of both household- and community-level WASH conditions. Supplementary interviews and observations were conducted in cases where questionnaire data were incomplete to ensure full coverage. The resulting mixed-method dataset served as the empirical foundation for the subsequent multilevel analyses.

Given that households were nested within communities, a multilevel analytical framework was employed to account for intra-community correlations in both sample-size adjustment and statistical modeling. A multilevel generalized linear model was fitted with infection rate as the dependent variable and WASH indicators as predictor variables, including two-way interaction terms to capture potential synergistic effects. Rather than employing a classical experimental RSM, response-surface analysis was conducted using model-predicted values derived from the fitted multilevel model to visualize nonlinear interactions and identify optimal intervention conditions. Minitab 21.0 was employed for generating response-surface visualizations and optimization results. This integrated analytical design provides a robust and context-appropriate framework for understanding WASH-related health challenges and advancing progress toward Sustainable Development Goal 6 (Clean Water and Sanitation).

### 2.2 Study area and population

**Fig. 2:**
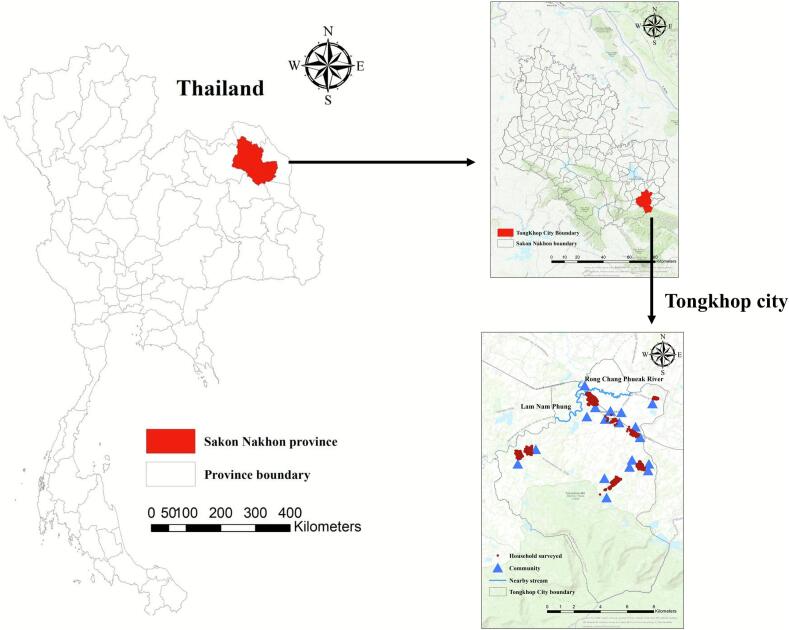
Study area: Tongkhop city, Sakon Nakhon province, northeastern Thailand.

The study was conducted in 18 communities located within Tongkhop city, Sakon Nakhon Province, in northeastern Thailand (Fig. 2). This area is recognized as a high-risk zone for fecal-pathogen infections, particularly *Opisthorchis viverrini*, due to persistent sanitation challenges, reliance on on-site fecal-sludge (FS) systems, and traditional raw-fish consumption practices. The study area represents an ecologically sensitive semi-rural municipality characterized by mixed land use, including residential settlements, rice cultivation, and aquaculture ponds. These environmental features create multiple human–environment interfaces that increase pathogen exposure through shared water resources and sanitation systems.

Community selection followed a purposive approach based on surveillance data from the Department of Disease Control (DDC, 2024), which identified consistently elevated infection rates in the study area. The selected communities represented a range of sanitation service coverage levels, population densities, and water-access conditions to ensure adequate heterogeneity for comparative analysis. Within each community, household sampling was conducted using a systematic random-sampling procedure based on official registries maintained by village health volunteers. If a selected household was vacant, non-residential, or declined participation, it was replaced by the next eligible household on the list to minimize nonresponse bias. Eligible participants were adults aged 18 years or older who had resided in the household for at least six months and provided informed consent prior to participation. Either the household head or primary caregiver served as the main respondent to ensure the accuracy and reliability of household-level WASH information.

Data were collected from 520 households across 18 communities in Tongkhop city. The required sample size was estimated using the standard single-proportion (prevalence) formula:

$$n\;=\;\frac{Z^{2}\cdot p\cdot \left(1‐p\right)}{E^{2}}$$

This approach is appropriate in our study because the primary outcome is an infection rate/proportion (i.e., prevalence expressed as cases per 1000 population), and the objective of this step was to ensure adequate precision for estimating that proportion. We recognize that this formula is intended specifically for estimating a rate/proportion (prevalence) and is not applicable for objectives such as estimating a mean, comparing means, or other analytic designs [26].

In the above equation, *Z* = 1.96 for a 95 % confidence level, *p* = 0.25 is the expected prevalence of fecal-pathogen infection, and E = 0.05 the desired margin of error. This yielded an initial simple-random-sample estimate of approximately 350 households. Because households were clustered within communities, we inflated the sample size using a design effect to account for within-community correlation:

$$D_{\mathrm{eff}}=1+\left(m-1\right)\rho$$

where m is the average number of households sampled per community and ρ is the intra-class correlation coefficient (ICC). Based on prior WASH studies conducted in similar settings (ρ ≈ 0.03–0.05), a design effect (D_eff_) of 1.5 was applied, resulting in a final target sample size of approximately 520 households.

### 2.3 Data collection

The data collection process integrated both primary and secondary sources to comprehensively assess unsafe WASH practices and their contribution to fecal pathogen infections under the environmental dimension of the One Health framework. Secondary data on infection prevalence and the status of unsafely managed fecal sludge were obtained from the Subdistrict Health Promotion Hospital in Tongkhop city and the national surveillance dataset of the Department of Disease Control (DDC, 2023). These data provided baseline indicators for community-level infection rates and sanitation service coverage.

Primary data were collected from May to August 2023 through a combination of structured household surveys, key informant interviews, and structured field observations. Collaboration with local public health officers, environmental sanitation staff, and village health volunteers facilitated coordinated data collection and improved the accuracy of contextual environmental assessments. Fieldwork was conducted under the supervision of the research teams from Thammasat University and Ramkhamhaeng University, and all enumerators received preparatory training on WASH indicators, interview techniques, and the use of standardized checklists and GPS equipment.

Household surveys gathered quantitative information on water access, sanitation systems, fecal sludge management, hygiene practices, and household demographics. Field observations followed a structured, non-participant, and overt approach to ensure transparency and minimize observer bias. Enumerators used standardized checklists to document sanitation and environmental conditions, including wastewater discharge points, cleanliness of facilities, and open drainage or potential contamination sources around households.

Key informant interviews were conducted with public health officers, environmental sanitation personnel, and community water-supply operators to gain insights into local management practices, barriers to sanitation improvement, and intersectoral collaboration in WASH governance. Qualitative findings were analyzed thematically and triangulated with quantitative survey data to validate results.

Data collection focused on three WASH domains: (1) access to safe water, (2) sanitation facilities and fecal sludge management, and (3) hygiene and food safety practices (Table S1). For water access, surveys recorded reliance on shared or untreated sources, treatment methods (boiling, filtration, or chlorination), and storage practices that may cause secondary contamination. Sanitation data included the types and condition of cesspools or septic tanks, frequency of desludging, and unsafe disposal behaviors. Hygiene and food-safety information were obtained through structured interviews and observation of handwashing behavior, soap availability, and raw-fish consumption habits.

All surveys and interviews were conducted in Thai, the participants' native language. Responses were recorded manually without audio or video recording. Participation was entirely voluntary. Before each interview, enumerators explained the study objectives, confidentiality procedures, and the participant's right to decline or withdraw at any time. Verbal consent was obtained because the study posed no physical or psychological risk and did not collect personal identifiers or sensitive health information. Although formal Institutional Review Board (IRB) approval was not sought, the study involved only non-identifiable environmental and behavioral data without experimental or clinical procedures.

To ensure data reliability, enumerators followed standardized recording protocols, and field supervisors reviewed completed forms daily for accuracy and completeness. Each household was assigned a code and GPS coordinate to enable linkage with secondary datasets. This mixed-method, multilevel data-collection framework ensured completeness, consistency, and representativeness for subsequent statistical and response-surface analyses.

### 2.4 Analytical framework and modeling approach

Descriptive statistics, including mean, median, and standard deviation, were employed for preliminary analyses to assess the distribution and variability of WASH service indicators. Data analysis followed a structured, stepwise approach to evaluate and optimize the relationships between unsafe WASH practices and fecal-pathogen infection rates using the integrated multilevel modeling and RSM framework, as summarized in Table S2. The analysis comprised four sequential steps:

**Step 1 Defining objectives and variable framework**

In this step, the analytical framework was defined and key WASH variables were operationalized within the environmental dimension of the One Health framework. The primary objective was to quantitatively assess how deficiencies in WASH conditions contribute to variations in community-level infection prevalence in northeastern Thailand. The analysis focused on how sanitation systems, water management, and hygiene behaviors jointly influence community-level infection risks through shared environmental pathways. Variables were organized into three domains: access to safe water, sanitation and FSM, and hygiene and food-safety practices, with each indicator numerically coded for multilevel modeling. Community-level means were calculated to represent collective environmental and behavioral conditions for each of the 18 communities, enabling integration of household survey data, field observations, and secondary surveillance records within a unified One Health–WASH analytical structure.

Household-level survey data were aggregated to the community level to align with the scale of the outcome variable (infection prevalence). Because the number of surveyed households varied across communities, community-level indicators were calculated as means (i.e., proportions) based on the actual number of households surveyed in each community, rather than assuming equal sample sizes. Thus, each community-level mean represents the proportion of households within that specific community exhibiting a given WASH characteristic (e.g., water treatment, desludging frequency, or handwashing practices).

The analytical framework consisted of a random-intercept multilevel generalized linear model coupled with RSM. The outcome variable was community-level infection prevalence (cases per 1000 population). Fixed effects included community-level WASH indicators derived from household surveys (water access and treatment, sanitation and fecal sludge management, and hygiene and food-safety practices), as well as their two-way interaction terms.

**Step 2 Data integration**

Primary data collected through household surveys, key informant interviews, and field observations across 18 communities in Tongkhop city, Sakon Nakhon Province, were systematically integrated with secondary data on infection prevalence and FSM status obtained from the Subdistrict Health Promotion Hospital and the Department of Disease Control (DDC). This process aligned household and community-level information within a single analytical framework, ensuring consistency in variable definitions, measurement units, and spatial identifiers. Qualitative information derived from interviews and field observations was thematically summarized and cross-validated with quantitative survey data to enhance interpretation of WASH behaviors and environmental sanitation patterns, particularly the local reliance on untreated surface water, cesspool use, and raw-fish consumption. The resulting harmonized dataset represented a comprehensive community-level profile of WASH conditions and infection risks, forming a robust empirical foundation for subsequent multilevel and response-surface analyses.

**Step 3 Data analysis**

The relationships between selected WASH variables and community-level infection rates were analyzed using a multilevel generalized linear modeling framework integrated with response-surface analysis. Statistical modeling was first performed to identify significant predictors and interaction effects among key WASH domains, including water access, sanitation, and hygiene behaviors. The fitted multilevel model generated predicted infection rates under varying combinations of WASH indicators, and these model-predicted values were subsequently applied within the RSM framework to visualize and quantify nonlinear interactions and synergistic effects among predictors. This analytical integration of multilevel modeling with RSM enabled the identification of critical threshold regions where coordinated improvements across multiple WASH components yielded the greatest reductions in infection prevalence. Insights derived from the response-surface plots guided the delineation of evidence-based intervention thresholds and the optimization of strategies for community-scale WASH improvements.

**Step 4 Optimization of WASH interventions**

The final analytical step focused on identifying optimal conditions for reducing infection prevalence based on the response surface outcomes. The optimization process established quantitative thresholds for key WASH indicators, such as the minimum effective coverage of water treatment, FSM service frequency, and hygiene compliance, that corresponded to the greatest predicted reductions in infection rates. These optimized thresholds informed the formulation of targeted intervention strategies emphasizing improvements in water treatment accessibility, sanitation infrastructure, and behavior change programs promoting consistent handwashing and the avoidance of raw food consumption.

In addition to the quantitative modeling of survey data, qualitative information derived from field observations and key informant interviews was analyzed thematically. Observation notes were organized according to the three WASH domains (water access, sanitation/FSM, and hygiene–food safety) and summarized to identify recurring patterns such as maintenance behaviors, environmental contamination sources, and hygiene compliance levels. Interview responses were manually coded into categories reflecting perceived barriers, local management practices, and community awareness of WASH risks. These qualitative insights were triangulated with household survey results to validate and enrich the quantitative findings, ensuring contextual interpretation of the statistical outcomes.

## 3 Results and discussion

### 3.1 Descriptive statistics of WASH performance in the study area

Descriptive statistics of WASH performance across the study area are summarized in Table 1, which presents household level indicators aggregated to the community level (*n* = 18 communities, representing a total of 520 households). For each community, the percentages indicate the proportion of surveyed households exhibiting specific WASH characteristics. For example, the percentage of households relying on shared surface water sources or practicing handwashing before meals was calculated using the total number of households surveyed within each community (denominator = number of households in that community, totaling 520 households overall). The mean and standard deviation values shown in Table 1 therefore represent the distribution of these community level percentages across the 18 communities.

**Table 1:** Descriptive statistics of WASH performance in the study area (*n* = 18).

Variables	Mean	S.D.
Population	438	143
Households (number)	175	68
Reliance on shared natural water sources (%)	55	22
Households treating water before consumption (%)	22	9
**Type and usage of sanitation facilities**		
- Cesspools (%)	83	3
- Septic tanks (%)	15	4
**Fecal sludge management**		
- Safely managed FS (%)	37	8
- FS emptying (%)	34	16
**Hygiene and food safety practices**		
- Handwashing before meals (%)	32	12
- Consumption of raw or undercooked fish (%)	47	12

The results indicate that, on average, 55 % ± 44 % of households rely on shared surface water sources, while only 22 % ± 18 % treat water before consumption, indicating substantial variability and limited adoption of safe water practices across communities. Sanitation facilities are predominantly cesspools (83 % ± 6 %), whereas septic tanks are used by only 15 % ± 8 % of households. Safely managed fecal sludge accounts for 37 % ± 16 % of households, and 34 % ± 32 % report regular sludge emptying, highlighting pronounced heterogeneity in sanitation service provision. Handwashing before meals is practiced by 32 % ± 24 % of households, and nearly half (47 % ± 24 %) consume raw or undercooked fish. These findings underscore the urgent need for targeted interventions, including improved access to safe water, enhanced FSM services, and community-based hygiene and food safety promotion programs to mitigate health risks and support sustainable WASH improvements.

### 3.2 Synergistic effects of access to safe water on fecal pathogen prevalence

**Fig. 3:**
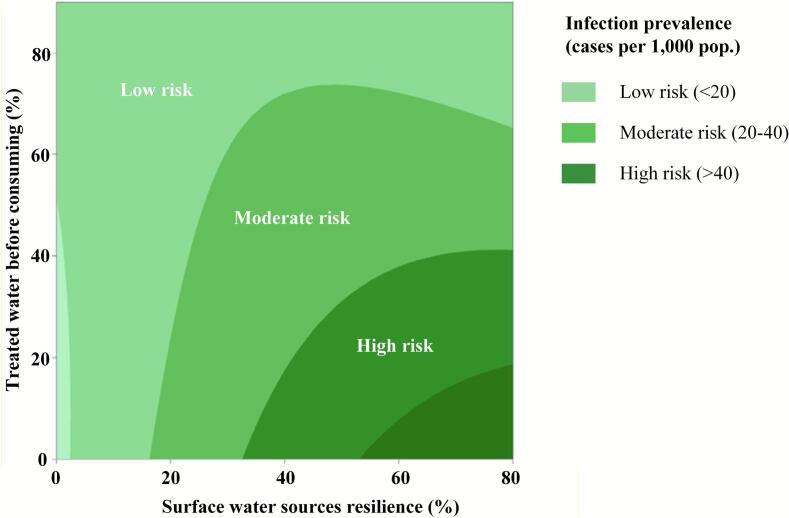
Contour plot of synergistic effects of access to safe water on fecal pathogen prevalence.

The contour plot (Fig. 3) illustrates the relationship between reliance on surface water sources (e.g., rivers, reservoirs, canals) and the proportion of households treating water before consumption, including methods such as boiling, sand filtration, and membrane filtration, mapped against infection prevalence rates. Risk zones are categorized as high (> 40 cases per 1000 population), moderate (20–40 cases per 1000 population), and low (< 20 cases per 1000 population), represented in varying shades of green, with darker shades indicating higher infection rates.

#### 3.2.1 High-risk zone (> 40 cases per 1000 population)

Households in high-risk zones rely heavily on untreated surface water sources, with over 50 % depending on them for daily consumption. However, fewer than 40 % implement water treatment before consumption, primarily through boiling, sand filtration, or household membrane filtration, thereby increasing susceptibility to fecal-pathogen infections [8]. Survey results also indicate a high prevalence of open defecation, exacerbating environmental contamination and facilitating fecal-oral pathogen transmission [11]. A critical concern in these zones is the limited awareness of fecal-pathogen infections, particularly helminth infections such as *Opisthorchis viverrini*. This knowledge gap contributes to persistent infection cycles and hinders effective disease-prevention interventions [12]. These findings align with studies by Ferreira et al. [17] and Paramita et al. [30], which highlight the strong association between inadequate sanitation management and high fecal-pathogen prevalence.

Short-term interventions such as implementing decentralized community-level water-treatment systems, including chlorination and slow-sand-filtration units, could enhance water safety [27]. Subsidizing household access to safe-water technologies, such as ceramic filtration and solar disinfection, can further enhance access to safe drinking water. Addressing open defecation through improved sanitation facilities and community-led total sanitation (CLTS) initiatives is crucial for reducing environmental contamination [39]. Long-term interventions should prioritize expanding access to safely managed water sources by investing in piped-water infrastructure, while ensuring adequate operation and maintenance of these systems and appropriate regulation of water-related costs (e.g., installation and purchase), as well as improving fecal sludge management systems [4]. Strengthening regulatory frameworks for safe wastewater and sludge disposal is also critical to preventing disease outbreaks in high-risk communities [45].

#### 3.2.2 Moderate-risk zone (20–40 cases per 1000 population)

Households in moderate-risk areas exhibit partial reliance on surface water sources (20–50 %) and inconsistent water-treatment practices (20–70 %), which compromise water quality and increase infection risks [2]. Survey results indicate that access to piped water is often intermittent in these zones, with water being shut off for 5–10 h per day or several times per month. This leads to fluctuating dependence on untreated or inadequately treated water sources. This inconsistency contributes to sporadic and ineffective household access to safe-water practices, increasing the risk of pathogen exposure. Although moderate-risk areas show lower rates of open defecation than high-risk zones, awareness and adherence to safe-water practices remain inconsistent. While knowledge of fecal-pathogen infections, particularly *Opisthorchis viverrini*, is more widespread, incomplete adoption of preventive measures continues to facilitate transmission cycles.

Mitigating infection risks requires a multifaceted approach integrating behavioral and infrastructural interventions. Household-water-treatment education programs should be introduced to encourage consistent purification practices, as targeted initiatives have been shown to mitigate microbial contamination in household drinking water [20]. Expanding access to cost-effective point-of-use (PoU) technologies such as biosand filters, chlorine-disinfection tablets, and ultraviolet (UV) purification devices offers sustainable solutions [17,21]. These technologies could enhance microbial water quality and reduce diarrheal disease incidence, particularly in resource-limited settings. Enhancing the reliability of piped-water infrastructure is essential to reducing dependence on untreated surface or groundwater sources. Investments in water-supply resilience will promote equitable access to safe drinking water and lessen reliance on unsafe alternatives [3].

Additionally, health-education campaigns should focus on fecal pathogen infections, especially *Opisthorchis viverrini*, and preventive behaviors such as safe food consumption and improved sanitation. Studies have shown that community-based educational efforts significantly improve knowledge retention and encourage behavioral change, leading to measurable reductions in *Opisthorchis viverrini* transmission [12]. Prakobwong et al. [32] demonstrated that a comprehensive One Health intervention, integrating human health education and treatment, management of reservoir hosts, and environmental control of snails and sanitation, substantially reduced *Opisthorchis viverrini* transmission across human, animal, and environmental domains in rural Thailand. Strengthening awareness of zoonotic-transmission pathways and promoting behavioral changes, such as avoiding raw or undercooked freshwater fish consumption, could further reduce the disease burden [31]. These integrated interventions, combining water quality improvements, infrastructure investment, and targeted health education, align with global efforts to achieve SDG 6 [35].

#### 3.2.3 Low-risk zone (<20 cases per 1000 population)

Households in low-risk zones demonstrate high rates of access to safe-water practices before consumption (> 70 %) and minimal reliance on surface-water sources (< 20 %), contributing to an infection prevalence of <20 cases per 1000 population. These findings highlight the critical role of improved water access, sanitation infrastructure, and compliance with hygiene and food-safety practices in reducing fecal-pathogen infections. The near absence of open defecation further minimizes contamination risks, while heightened awareness of fecal-sludge-related infections, particularly *Opisthorchis viverrini*, enhances preventive practices and disease-control efforts [21]. Sustaining these public-health gains requires continued investment in infrastructure maintenance and systematic monitoring. Even in low-risk areas, *peri*odic water-quality testing is essential for detecting microbiological and chemical contaminants, enabling early interventions to mitigate potential health risks. Strengthening regulatory frameworks and enforcement mechanisms for drinking-water safety further safeguards water quality and reduces exposure risks [25].

Additionally, promoting safe water storage combined with appropriate household water treatment is crucial for preventing secondary contamination, particularly in rural and peri urban areas where intermittent piped water supply requires prolonged water storage. This aligns with findings from GBD [19] and Nshimiyimana et al. [29], which indicate that improper storage practices, such as uncovered containers and the absence of residual disinfectants, facilitate microbial regrowth and introduce pathogens into otherwise treated water. Public health campaigns should therefore reinforce both safe storage practices and household level treatment methods, such as boiling or disinfection, especially in rural and peri urban settings where water supply reliability fluctuates.

The response surface analysis quantified a distinct nonlinear interaction between untreated-water reliance and household treatment rates. Infection prevalence remained above 40 cases per 1000 population when more than 50 % of households depended on untreated surface water and fewer than 40 % practiced treatment, but declined steeply once treatment adoption entered the 40–70 % band while untreated reliance fell below 50 %. This identifies a data-driven threshold region rather than a purely descriptive gradient: incremental gains below these cut-offs yield little reduction, whereas coordinated improvements across both variables shift communities from high- to moderate-risk. Such quantitative boundaries provide new evidence on the minimal effective coverage needed to cross epidemiological tipping points, information that was previously unavailable from conventional WASH surveys. The model therefore extends existing knowledge by transforming general recommendations into predictive thresholds that directly inform priority setting and resource allocation for SDG 6 implementation. These quantitative insights are summarized in Table S3, which translates the modeled thresholds into zone-specific intervention targets for decision-makers.

### 3.3 Synergistic effects of sanitation management on fecal pathogen prevalence

**Fig. 4:**
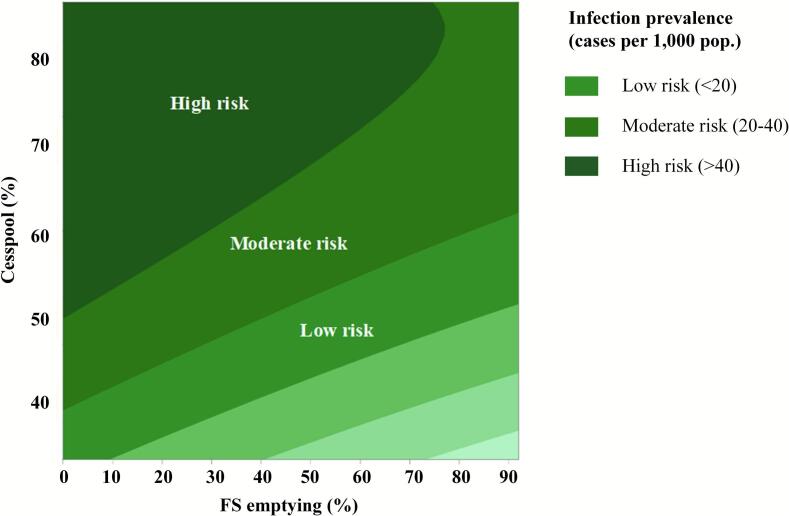
Contour plot of synergistic effects of sanitation management practices on fecal pathogen prevalence.

Sanitation management plays a critical role in controlling the prevalence of fecal pathogen infections, such as *Opisthorchis viverrini*, a major public health concern in Southeast Asia [6]. Poor FSM and high reliance on rudimentary sanitation systems, such as cesspools, contribute to persistent contamination of water sources [14]. Expanded sanitary toilet coverage and safe sanitation, for example, significantly lowered the incidence of *brucellosis*, *echinococcosis*, and *leptospirosis* by interrupting pathogen cycles shared between humans, animals, and contaminated environments [15,18,46]. These studies collectively demonstrated that sanitation infrastructure is not only a human-health intervention but also a mechanism for reducing pathogen survival and mobility across interconnected ecological compartments. By minimizing the deposition of fecal pathogens into soil, surface water, and agricultural landscapes, improved containment and fecal treatment reduces the environmental pathogen loads that otherwise allow zoonotic agents to circulate among animal hosts and spill over to humans. This study examines the influence of FSM frequency and cesspool prevalence on fecal pathogen infection rates, utilizing a contour plot to visualize risk zones and propose evidence-based interventions. Fig. 4 illustrates the correlation between FSM frequency, cesspool reliance, and infection prevalence, categorizing infection risks into high (>40 cases per 1000 population), moderate (20–40 cases per 1000 population), and low (<20 cases per 1000 population) zones.

#### 3.3.1 High-risk zone (> 40 cases per 1000 population)

Areas in this zone exhibit over 50 % reliance on cesspools and low FSM frequency (<10 %), leading to severe environmental contamination and high fecal pathogen infections [23]. To mitigate this, a mandatory desludging program should be implemented, ensuring cesspools are emptied at least once a year. Expanding FSM facilities, such as vacuum trucks and FS treatment plants, is essential for improving service accessibility. Local governments should invest in mobile FSM services to reach underserved communities. Additionally, public health awareness campaigns should educate communities on the risks of improper sludge disposal and the importance of safe sanitation practices. Implementing these interventions collectively can significantly reduce pathogen exposure and infection rates in high-risk areas [7].

#### 3.3.2 Moderate-risk zone (20–40 cases per 1000 population)

Moderate-risk areas rely on cesspools (40–50 %) with FSM occurring at moderate rates (10–40 %). Despite some FSM interventions, inconsistent desludging and inadequate sanitation infrastructure continue to pose risks. Establishing community-managed FSM programs, in which local authorities and residents collaborate on proper sludge disposal, can enhance sanitation outcomes. Decentralized on-site treatment systems, such as biodigesters and constructed wetlands, offer effective alternatives, reducing sludge accumulation and contamination [3]. Additionally, subsidized FSM services should be introduced, particularly for low-income households, to encourage regular desludging and eliminate economic barriers to sanitation access. These interventions aim to improve FSM efficiency, reducing infection rates and preventing environmental contamination [18].

#### 3.3.3 Low-risk zone (<20 cases per 1000 population)

Low-risk areas exhibit cesspool reliance below 40 % and frequent FS emptying (> 40 %), which significantly reduces the prevalence of fecal-pathogen infections. While sanitation practices are improved, ongoing regulatory enforcement is necessary to sustain low infection rates. FSM service providers must comply with national sanitation guidelines to prevent lapses in waste management [22]. Integrating FSM with wastewater-treatment systems can facilitate safe sludge processing and prevent water-body recontamination. Expanding education programs on hygiene and food-safety practices, particularly in peri-urban and rural areas, is essential for reinforcing safe-sanitation behaviors [11]. Maintaining FSM efficiency and infrastructure will help sustain low fecal-pathogen infection rates, supporting SDG 6 and improving public-health outcomes in affected communities.

The response-surface analysis revealed a nonlinear interaction between cesspool reliance and FSM frequency that defines clear tipping points for infection reduction. Infection prevalence remained above 40 cases per 1000 population when cesspool reliance exceeded 50 % and FSM emptying was below 10 %. The model predicted a marked decline in prevalence once desludging frequency increased into the 35–40 % range and cesspool use decreased to 40–50 %. This synergy indicates that isolated improvements in FSM or cesspool replacement yield limited benefits, whereas simultaneous enhancement of both parameters moves communities from the high- to the moderate-risk zone. These data-driven thresholds therefore provide new evidence on the minimum service coverage required to achieve epidemiological impact and support cost-effective planning for FSM investments under SDG 6. The modeled cut-off conditions are summarized in Table S4, which translates these quantitative findings into zone-specific sanitation targets for policymakers.

### 3.4 Synergistic effects of hygiene and food safety practices on fecal pathogen prevalence

**Fig. 5:**
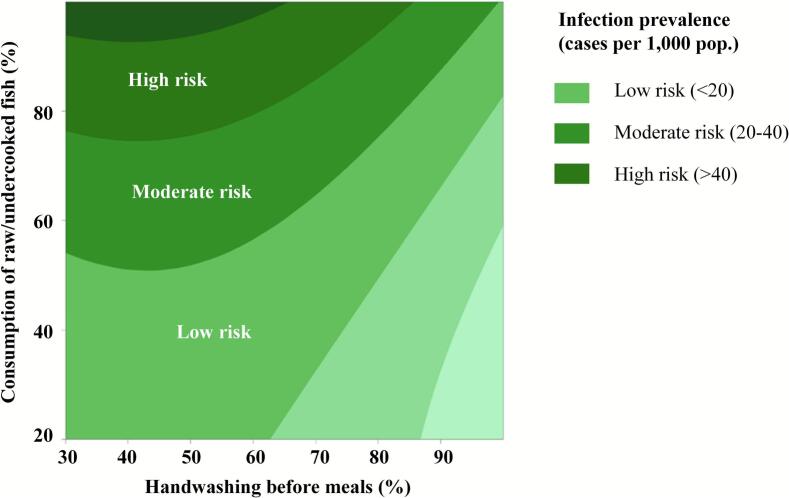
Contour plot of synergistic effects of hygiene and food safety practices on fecal pathogen prevalence.

The contour plot in Fig. 5 highlights the interplay between hygiene and food safety practices, specifically handwashing before meals and the consumption of raw or undercooked fish, and their collective impact on infection prevalence. The data reveal a strong correlation between these behaviors and fecal pathogen prevalence, emphasizing the necessity of multi-faceted interventions. The results indicate that while hand hygiene is an essential barrier against fecal-oral pathogen transmission, its effectiveness diminishes when exposure to high-risk foods remains uncontrolled. This underscores the need for integrated interventions that simultaneously promote behavioral changes in hygiene and food safety practices to achieve sustainable infection risk reduction.

#### 3.4.1 High-risk zone (> 40 cases per 1000 population)

In the high-risk zone, raw or undercooked fish consumption exceeds 80 %, and handwashing compliance is below 50 %, resulting in infection prevalence surpassing 40 %. These findings corroborate prior studies indicating that *Opisthorchis viverrini* and other foodborne parasites remain prevalent in populations with high raw fish consumption [9]. The combined effect of high-risk dietary habits and inadequate hand hygiene suggests that conventional health interventions, such as promoting hygiene and food safety practices alone, are insufficient.

A comprehensive approach is necessary to mitigate the high infection prevalence in this zone. Community-based awareness campaigns tailored to local dietary habits should emphasize the risks associated with raw fish consumption, as evidences suggest that behavioral change is more effective when culturally adapted [40]. Strengthening food safety regulations to enforce proper fish handling and preparation standards is crucial, as regulatory interventions have been shown to significantly reduce foodborne infections [24]. Expanding WASH programs to improve access to clean water and promote consistent handwashing behaviors can reduce fecal-oral transmission pathways [19]. Additionally, routine deworming and targeted screening for high-risk populations in endemic areas have been recommended to minimize long-term health impacts.

#### 3.4.2 Moderate-risk zone (20–40 cases per 1000 population)

The moderate-risk zone includes populations with fish consumption rates between 50 and 80 % and handwashing compliance ranging from 50 to 80 %. Despite improved hygiene and food safety practices, infection prevalence remains high. This suggests that while handwashing mitigates risk, it does not fully compensate for the exposure to foodborne pathogens, highlighting the limitations of hygiene interventions when dietary risk factors persist [24,36].

For populations within this zone, promoting safe cooking techniques such as boiling or frying fish thoroughly can substantially reduce pathogen exposure [35]. School-based hygiene and food safety education programs should be reinforced to instill lifelong handwashing habits, as early interventions have proven to be effective in reducing disease transmission in the long term [1]. Furthermore, strengthening household sanitation by improving waste disposal and water quality can prevent the indirect spread of fecal-oral pathogens [34].

#### 3.4.3 Low-risk zone (<20 cases per 1000 population)

In the low-risk zone, where handwashing compliance exceeds 80 % and raw fish consumption is below 50 %, infection prevalence remains under 20 %. This demonstrates the synergistic effects of proper hygiene, food safety practices, and dietary modifications in minimizing exposure to fecal pathogens. To sustain low infection rates, continued public health campaigns reinforcing hygiene and safe food handling practices are necessary [33]. Routine health monitoring can identify emerging risks and enable timely interventions, thereby preventing potential outbreaks. Integrating hygiene and food safety education into schools and community programs will help ensure long-term compliance with best practices [38].

The findings of this study highlight the necessity of targeted interventions to mitigate infection risk based on behavioral factors as presented in Table S5. Therefore, a comprehensive intervention incorporating regulatory, educational, and medical interventions is essential to achieve sustained reductions in infection prevalence and support SDG 6 goals of improving sanitation and public health outcomes. This study demonstrates how environmental and behavioral dimensions of the One Health framework can be applied to community-scale WASH improvement. By integrating household-level data with environmental sanitation indicators, the findings highlight the importance of intersectoral collaboration among public health, local environmental agencies, and community water-supply systems. Such coordination can strengthen ecosystem resilience, reduce exposure to fecal pathogens, and sustain long-term health benefits without requiring direct animal surveillance data.

The novelty of this research lies not in reiterating established WASH principles but in quantifying the marginal gains achievable from specific combinations of hygiene and food-safety behaviors. The response-surface analysis revealed a nonlinear interaction between handwashing compliance and raw-fish consumption that defines measurable behavioral thresholds. Infection prevalence remained above 40 cases per 1000 population when handwashing compliance was below 50 % and raw-fish consumption exceeded 80 %, but it declined sharply once handwashing reached 80 % and raw-fish intake dropped below 50 %. These findings demonstrate that hygiene interventions alone are insufficient unless accompanied by dietary modification. By modeling these nonlinear interactions, the study provides a predictive decision-support framework that enables policymakers to allocate resources efficiently, for example by prioritizing hygiene promotion in communities already practicing safer diets or focusing on dietary risk reduction where hygiene compliance has plateaued.

### 3.5 Strengths and limitations of the study

This study has several strengths. It employed an integrated, multilevel approach that combined both primary (household surveys, interviews, and observations) and secondary (disease-surveillance and FSM service) data, allowing for comprehensive analysis across WASH domains. The use of multilevel modeling and response-surface analysis enabled quantification of nonlinear and synergistic interactions among WASH variables, enhancing the predictive and policy relevance of the findings. However, this study also has limitations. The cross-sectional design restricts causal inference, as data were collected at a single point in time. Secondary infection data were derived from existing surveillance reports, which may underreport subclinical or asymptomatic cases. In addition, behavioral observations could be affected by social desirability bias, potentially overestimating hygiene compliance. Future longitudinal or intervention-based studies could strengthen causal understanding and validate the predictive thresholds identified in this research.

## 4 Conclusions

This study highlights the significant role of WASH practices in shaping the prevalence of fecal-pathogen infections in northeastern Thailand, particularly in Tongkhop city, Sakon Nakhon Province. By integrating multilevel modeling with response-surface analysis, this research provides a data-driven understanding of nonlinear interactions among key WASH components and identifies quantitative thresholds that influence infection rates. The findings reveal that infection prevalence exceeds 40 cases per 1000 population in high-risk areas where households primarily rely on untreated water sources, have inefficient sanitation systems, and show low compliance with hygiene and food-safety practices. In contrast, low-risk zones, characterized by improved access to safe water, regular FSM, and higher adherence to hygiene and food-safety standards, report significantly lower infection rates.

To effectively reduce fecal-pathogen transmission, both short-term and long-term strategies are required. High-risk areas need urgent actions such as expanding decentralized water treatment, ensuring regular fecal-sludge emptying, and implementing targeted hygiene and food-safety education campaigns. For moderate-risk zones, strengthening community-led water and sanitation programs and promoting safe-food practices are critical for reducing exposure. In low-risk areas, sustaining progress through regular infrastructure maintenance, continuous monitoring, and strict regulatory enforcement is essential to preserve health gains.

Overall, this study reinforces the importance of integrating environmental management with public health and behavioral interventions under the One Health framework. Strengthening collaboration among health authorities, environmental agencies, and community water-supply systems is essential to sustain long-term improvements in WASH conditions, enhance ecosystem resilience, and reduce fecal pathogen transmission in alignment with SDG 6.

## CRediT authorship contribution statement

**Achara Taweesan:** Writing – original draft, Methodology, Investigation, Funding acquisition, Formal analysis, Conceptualization. **Thammarat Koottatep:** Writing – review & editing. **Thongchai Kanabkaew:** Writing – original draft, Methodology, Formal analysis, Conceptualization. **Rathanit Sukthanapirat:** Writing – review & editing. **Chongrak Polprasert:** Writing – review & editing.

## Declaration of generative AI and AI-assisted technologies in the writing process

During the preparation of this manuscript, the author used ChatGPT (developed by OpenAI) solely to assist with spelling and grammar checking. After using this tool, the author critically reviewed, revised, and approved the final version of the manuscript and takes full responsibility for its content.

## Declaration of competing interest

The authors state that they have no conflicts of interest.

## Data Availability

The authors do not have permission to share data.

## Appendix A Supplementary data

Supplementary material Image 1

## References

[1] Abdulhadi R., Bailey A., Van Noorloos F. (2024). Access inequalities to WASH and housing in slums in low- and middle-income countries (LMICs): a scoping review. Glob. Public Health.

[2] Begum M.R., Al Banna M.H., Akter S., Kundu S., Sayeed A., Hassan M.N., Chowdhury S., Khan M.S.I. (2020). Effectiveness of WASH education to prevent diarrhea among children under five in a community of Patuakhali, Bangladesh. SN Compr. Clin. Med..

[3] Blasi S., Ganzaroli A., Noni I.D. (2022). Smartening sustainable development in cities: strengthening the theoretical linkage between smart cities and SDGs. Sustain. Cities Soc..

[4] Blokus-Dziula A., Dziula P., Kamedulski B., Michalak P. (2023). Operation and maintenance cost of water management systems: analysis and optimization. Water.

[5] Boonjaraspinyo S., Boonmars T., Ekobol N., Artchayasawat A., Sriraj P., Aukkanimart R., Pumhirunroj B., Sripan P., Songsri J., Juasook A., Wonkchalee N. (2023). Prevalence and associated risk factors of intestinal parasitic infections: a population-based study in Phra Lap Sub-District, Mueang Khon Kaen District, Khon Kaen Province, Northeastern Thailand. Trop. Med. Infect. Dis..

[6] Capone D., Berendes D.M., Cumming O., Holcomb D.A., Knee J., Konstantinidis K.T., Levy K., Nalá R., Risk B., Brown J. (2021). Impact of an urban sanitation intervention on enteric pathogen detection in soils. Environ. Sci. Technol..

[7] Centers for Disease Control and Prevention (2024). Clinical Overview of *Opisthorchis*. https://www.cdc.gov/liver-flukes/hcp/clinical-overview-opisthorchis/index.html.

[8] Cetrulo T., Marques R., Cetrulo N., Malheiros T. (2020). Monitoring inequality in water access: challenges for the 2030 agenda for sustainable development. Sci. Total Environ..

[9] Charoensuk L., Chedtabud K., Chaipibool S., Laothong U., Suwannatrai A., Pinlaor S., Prakobwong S. (2024). Integrated one-health approach for prevention and control of *Opisthorchis viverrini* infection in rural Thailand: a 3-year study. Parasitol. Res..

[10] Chaúque B.J.M., Issufo M., Benitez G.B., Cossa V.C., Chaúque L.G.H., Stauber C.E., Benetti A.D., Rott M.B. (2023). Why do low-cost point-of-use water treatment technologies succeed or fail in combating waterborne diseases in the field? A systematic review. J. Environ. Chem. Eng..

[11] Chowdhury M.A., Nowreen S., Tarin N.J., Hasan M.R., Zzaman R.U., Amatullah N.I. (2022). WASH and MHM experiences of disabled females living in Dhaka slums of Bangladesh. J. Water Sanit. Hyg. Dev..

[12] Crellen T., Sithithaworn P., Pitaksakulrat O., Khuntikeo N., Medley G.F., Hollingsworth T.D. (2021). Towards evidence-based control of *Opisthorchis viverrini*. Trends Parasitol..

[13] Department of Disease Control (2023). Guideline for the Prevention and Control of Disease and Health Hazards. https://ddc.moph.go.th/dsp.

[14] Devane M.L., Moriarty E., Weaver L., Cookson A., Gilpin B. (2020). Fecal indicator bacteria from environmental sources; strategies for identification to improve water quality monitoring. Water Res..

[15] Dickin S., Dagerskog L., Dione M., Thomas L., Arcilla J. (2025). Towards a one health approach to WASH to tackle zoonotic disease and promote health and wellbeing. PLOS Water.

[16] Edefo J.W. (2025). Water and sanitation access in Nigeria. Water Conserv. Sci. Eng..

[17] Ferreira D.C., Graziele I., Marques R.C., Gonçalves J. (2021). Investment in drinking water and sanitation infrastructure and its impact on waterborne diseases dissemination: the Brazilian case. Sci. Total Environ..

[18] Fuhrmeister E.R., Ercumen A., Pickering A.J., Jeanis K.M., Crider Y., Ahmed M., Brown S., Alam M., Sen D., Islam S., Kabir M.H., Rahman M., Kwong L.H., Arnold B.F., Luby S.P., Colford J.M., Nelson K.L. (2020). Effect of sanitation improvements on pathogens and microbial source tracking markers in the rural Bangladeshi household environment. Environ. Sci. Technol..

[19] GBD (2023). Global, regional, and national burden of diabetes from 1990 to 2021, with projections of prevalence to 2050: a systematic analysis for the global burden of disease study 2021. Lancet.

[20] John C.K., Ajibade F.O. (2024). Exploring the dynamics of WASH services: challenges, enablers, and strategies for improvement. Discov. Civ. Eng. J..

[21] Knee J., Sumner T., Adriano Z., Anderson C., Bush F., Capone D., Casmo V., Holcomb D., Kolsky P., MacDougall A., Molotkova E., Braga J.M., Russo C., Schmidt W.P., Stewart J., Zambrana W., Zuin V., Nalá R., Cumming O., Brown J. (2021). Effects of an urban sanitation intervention on childhood enteric infection and diarrhea in Maputo, Mozambique: a controlled before-and-after trial. eLife.

[22] Kwong L.H., Sen D., Islam S., Shahriar S., Benjamin-Chung J., Arnold B.F., Hubbard A., Parvez S.M., Islam M., Unicomb L., Rahman MdM, Nelson K., Colford J.M., Luby S.P., Ercumen A. (2021). Effect of sanitation improvements on soil-transmitted helminth eggs in courtyard soil from rural Bangladesh: evidence from a cluster-randomized controlled trial. PLoS Negl. Trop. Dis..

[23] Lapat J.J., Opee J., Apio M.C., Akello S., Ojul C.L., Onekalit R., Francis O.J., Lalweny D., Latigo K.J.P., Lebu S., Ochola E., Bongomin F. (2024). A one health approach toward the control and elimination of soil-transmitted helminthic infections in endemic areas. IJID One Health.

[24] Lin A., Ali S., Arnold B.F., Rahman M.Z., Alauddin M., Grembi J., Mertens A.N., Famida S.L., Akther S., Hossen M.S., Mutsuddi P., Shoab A.K., Hussain Z., Rahman M., Unicomb L., Ashraf S., Naser A.M., Parvez S.M., Ercumen A., Benjamin-Chung J., Haque R., Ahmed T., Hossain M.I., Choudhury N., Jannat K., Alauddin S.T., Minchala S.G., Cekovic R., Hubbard A.E., Stewart C.P., Dewey K.G., Colford J.M., Luby S.P. (2020). Effects of water, sanitation, handwashing, and nutritional interventions on environmental enteric dysfunction in young children: a cluster-randomized, controlled trial in rural Bangladesh. Clin. Infect. Dis..

[25] Nachaiwieng W., Sanit S., Kongta N., Saingamsook J., Duangmano S., Pornprasert S., Somboon P., Yanola J. (2024). The impact of an integrated intervention program combining drug therapy with water, sanitation, and hygiene (WASH) education on reinfection with intestinal parasitic infections among the Karen hill tribe in northern Thailand. Parasit. Vectors.

[26] Naing L., Nordin R.B., Abdul Rahman H., Naing Y.T. (2022). Sample size calculation for prevalence studies using Scalex and ScalaR calculators. BMC Med. Res. Methodol..

[27] Nelson S., Drabarek D., Jenkins A., Negin J., Abimbola S. (2021). How community participation in water and sanitation interventions impacts human health, WASH infrastructure and service longevity in low-income and middle-income countries: a realist review. BMJ Open.

[28] Nowreen S., Chowdhury M.A., Tarin N.J., Hasan M.R., Zzaman R.U. (2022). A participatory SWOT analysis on water, sanitation, and hygiene management of disabled females in Dhaka slums of Bangladesh. J. Water Sanit. Hyg. Dev..

[29] Nshimiyimana L., Mbituyumuremyi A., Ower A., Mbonigaba J.B., Palacio K., Musarurwa C., Uwizeye J., Hitiyaremye N., Tuyishime A., Huston T., Nyandwi E., Rujeni N., Ruberanziza E. (2025). Water and sanitation factors associated with schistosomiasis and soil transmitted helminthiasis persistence in Rwanda. Sci. Rep..

[30] Paramita N., Purwana R., Hartono D.M., Soesilo T.E.B. (2025). Determining factors and strategy in sustainable fecal sludge management services. Groundw. Sustain. Dev..

[31] Pengput A., Schwartz D.G. (2020). Risk factors for *Opisthorchis Viverrini* infection: a systematic review. J. Infect. Public Health.

[32] Prakobwong S., Charoensuk L., Chaipibool S., Chedtabud K., Laothong U., Suwannatrai A.T., Blair D., Pinlaor S. (2025). One health integrated strategies for sustainable control of *Opisthorchis viverrini* infections in rural endemic areas of Thailand. Infect. Dis. Poverty.

[33] Quattrochi J.P., Coville A., Mvukiyehe E., Dohou C.J., Esu F., Cohen B., Bokasola Y.L., Croke K. (2021). Effects of a community-driven water, sanitation and hygiene intervention on water and sanitation infrastructure, access, behaviour, and governance: a cluster-randomised controlled trial in rural Democratic Republic of Congo. BMJ Glob. Health.

[34] Romano G., Ferreira D., Marques R., Carosi L. (2020). Waste services’ performance assessment: the case of Tuscany, Italy. Waste Manag..

[35] Sadoff C.W., Borgomeo E., Uhlenbrook S. (2020). Rethinking water for SDG 6. Nat. Sustainability.

[36] Saroj S.K., Goli S., Rana M.J., Choudhary B.K. (2020). Availability, accessibility, and inequalities of water, sanitation, and hygiene (WASH) services in Indian metro cities. Sustain. Cities Soc..

[37] Sasidharan R., Kumar A. (2022). Response surface methodology for optimization of heavy metal removal by magnetic biosorbent made from anaerobic sludge. J. Indian Chem. Soc..

[38] Shoesmith A., Hall A., Wolfenden L., Shelton R.C., Powell B.J., Brown H., McCrabb S., Sutherland R., Yoong S., Lane C., Booth D., Nathan N. (2021). Barriers and facilitators influencing the sustainment of health behavior interventions in schools and childcare services: a systematic review. Implement. Sci..

[39] Singh S., Raju N.J., Mehmood G., Gupta S.K., Ahmed S. (2024). A review of the current scenario and best possible solution for fecal sludge management (FSM) in India. Groundw. Sustain. Dev..

[40] Singh S., Kriti M., Anamika K.S., Sharma P., Pal N., Sarma D.K., Tiwari R., Kumar M. (2025). A one health approach addressing poultry-associated antimicrobial resistance: human, animal and environmental perspectives. Microbe.

[41] UNICEF (2022).

[42] Veza I., Spraggon M., Fattah I.M.R., Idris M. (2023). Response surface methodology (RSM) for optimizing engine performance and emissions fueled with biofuel: review of RSM for sustainability energy transition. Results Eng..

[43] Wattanawong O., Iamsirithaworn S., Kophachon T., Nak-Ai W., Wisetmora A., Wongsaroj T., Dekumyoy P., Nithikathkul C., Suwannatrai A.T., Sripa B. (2021). Current status of helminthiases in Thailand: a cross-sectional, nationwide survey, 2019. Acta Trop..

[44] WHO (2021).

[45] Wolf J., Hubbard S., Brauer M., Ambelu A., Arnold B.F., Bain R. (2022). Effectiveness of interventions to improve drinking water, sanitation, and handwashing with soap on risk of diarrhoeal disease in children in low-income and middle-income settings: a systematic review and meta-analysis. Lancet.

[46] Zhao S., Ma J., Li W. (2025). Flushing away disease: a one health perspective on sanitation, pathogen transmission, and rural health in China. One Health.

