# Supplementary material for: A data-driven approach for mitigation of fecal pathogen infections from unsafe WASH practices
Source: One Health. 2026 Jan 3;22:101317. doi: 10.1016/j.onehlt.2026.101317 (PMC12811417; doi:10.1016/j.onehlt.2026.101317)
Supplement: Supplementary file 1 — Supplementary material [file mmc1.docx]

**Supplementary data**

**Table S1** Summarizing data collection methods in this study

| **WASH component** | **Focus area** | **Methods Used** | **Key data collected** |
| --- | --- | --- | --- |
| **Primary data** |  |  |  |
| Access to safe water | - Water source types  - Water treatment methods  - Water storage practices | - Household surveys  - Structured interviews  - Field observations | - Types of water sources  - Water treatment methods used  - Prevalence of shared water source usage  - Risk of contamination from storage practices |
| Sanitation facilities | - Availability and  accessibility  - Maintenance and  FSM practices | - Household surveys  - Structured interviews  - Field observations | - Types and condition of sanitation facilities  - Prevalence of open defecation  - Frequency of septic tank emptying  - FS disposal methods  - Proximity of sanitation facilities to water sources |
| Fecal sludge management (FSM) | - Safe disposal and management of FS | - Field observations | - Proportion of safely managed vs. unsafely discharged FS  - Physical condition of sanitation infrastructure |
| Hygiene and food safety practices | - Handwashing practices  - Cultural behaviors increasing pathogen exposure  - Community awareness on hygiene and food safety practices | - Structured surveys  - Interviews  - Field observations | - Frequency and adherence to handwashing with soap  - Availability of handwashing stations  - Consumption of raw/undercooked food  - Community engagement in hygiene and food safety practices education initiatives |
| **Secondary data** |  |  |  |
| Prevalence of fecal pathogen infections and FSM practices | - Baseline statistics on infection rates  - Sanitation mismanagement trends | - Data sourced from the Sub-district Health Promotion Hospital in Tongkhop city  - A nationwide survey conducted by the Department of Disease Control during the period from 2017 to 2023 | - Infection prevalence rates  - Percentage of unsafely managed FS |

**Table S2** A step-by-step guide to applying RSM for data analysis

| **Step** | **Description** | **Expected Outcome** |
| --- | --- | --- |
| 1.Defining objectives and variable framework | Identify how inadequate access to safe water, poor sanitation infrastructure, and unhygienic behaviors contribute to infection rates | Primary focus areas: water access, sanitation infrastructure, and hygiene and food safety practices |
| 2. Data integration | Combine primary data (surveys, interviews, observations) and secondary data (infection rates, FSM practices) | Comprehensive dataset representing infection rates and key WASH variables for analysis |
| 3. Data analysis | Analyze the relationships between WASH variables and infection rates to identify significant patterns and interactions | Insights into the relative and combined impacts of WASH practices on infection rates |
| 4. Optimization | Use RSM to determine critical thresholds and optimal conditions for reducing infection rates. | Optimal interventions for improving water access, sanitation infrastructure, and hygiene and food safety practices |

**Table S3** Targeted interventions on access to safe water to mitigate infection risks

| **Risk zone** | **Surface water sources reliance** | **Treated water before consumption** | **Infection prevalence**  **(cases per 1,000 pop.)** | **Specific interventions** |
| --- | --- | --- | --- | --- |
| High risk | > 50% | < 40% | >40 | - Deploy community water purification systems (chlorination, filtration)  - Expand access to safe drinking water through decentralized treatment  - Provide subsidized household treatment (SODIS, ceramic filters) |
| Moderate risk | 20-50% | 40-70% | 20-40 | - Strengthen community-based programs to promote consistent use of safe water treatment techniques (e.g., boiling, chlorination, filtration)  - Increase affordability and accessibility of point-of-use (PoU) treatment (e.g., biosand filters, chlorine tablets, UV purification)  - Improve the reliability of piped water infrastructure to enhance water supply continuity and reduce reliance on untreated sources  - Reinforce awareness programs by emphasizing fecal sludge transmission risks and safe food handling practices |
| Low risk | < 20% | > 70% | < 20 | - Regularly inspect and maintain of water supply systems to prevent contamination risks - Conduct periodic water quality testing within distribution networks and household storage facilities - Promote covered storage containers, regular cleaning, and the use of proper residual disinfectants to prevent contamination |

**Table S4** Targeted interventions in sanitation management practices to mitigate infection risk

| **Risk zone** | **Cesspool**  **(%)** | **FS emptying (%)** | **Infection prevalence**  **(cases per 1,000 pop.)** | **Specific interventions** |
| --- | --- | --- | --- | --- |
| High risk | >50% | <10% | >40 | - Increase FS emptying to ≥40% (at least once a year)  - Expand FSM facilities (e.g. vacuum trucks, FS treatment facilities)  - Subsidize FSM services to improve accessibility  - Alternative sanitation solutions (biogas digesters, composting toilets) |
| Moderate risk | 40-50% | 10-40% | 20-40 | - Decentralized FSM solutions (community treatment plants, anaerobic digesters)  - Flexible desludging fees to increase participation  - Integration with wastewater treatment to ensure safe disposal  - Regulatory enforcement to improve FSM compliance |
| Low risk | < 40% | >40% | < 20 | - Upgrade FSM facilities to meet future demand  - Real-time FSM monitoring to track desludging compliance  - Ongoing hygiene and food safety education programs to reinforce safe sanitation behaviors |

**Table S5** Targeted interventions on hygiene and food safety practices to mitigate infection risks

| **Risk zone** | **Raw or undercooked fish consumption** | **Handwashing**  **before meals** | **Infection prevalence**  **(cases per 1,000 pop.)** | **Specific interventions** |
| --- | --- | --- | --- | --- |
| High risk | >80% | <50% | >40 | - Implement community-based awareness programs to discourage raw fish consumption  - Strengthen food safety regulations to ensure proper handling and preparation of fish  - Enhance WASH programs to improve handwashing compliance  - Introduce routine deworming and screening for high-risk populations |
| Moderate risk | 50-80% | 50-80% | 20-40 | - Promote safe cooking techniques to mitigate partial or undercooked fish consumption  - Implement school-based hygiene education programs to improve handwashing practices  - Strengthen household-level sanitation to prevent fecal-oral pathogen transmission |
| Low risk | < 50% | >80% | < 20 | - Maintain hand hygiene standards through continuous public health campaigns  - Conduct periodic health monitoring to detect emerging infection risks  - Ensure the sustained integration of hygiene and food safety education into school and community programs |
